# Impacts of AD-Related *ABCA7* and *CLU* Variants on Default Mode Network Connectivity in Healthy Middle-Age Adults

**DOI:** 10.3389/fnmol.2020.00145

**Published:** 2020-07-31

**Authors:** Xin Yuan Zhang, Yun Fei Wang, Li Juan Zheng, Han Zhang, Li Lin, Guang Ming Lu, Long Jiang Zhang

**Affiliations:** Affiliated Jinling Hospital, Medical School of Nanjing University, Nanjing, China

**Keywords:** ATP-binding cassette A7, clusterin, default mode network, Alzheimer’s disease, middle-age adults

## Abstract

**Objective:**

To investigate the impact of Alzheimer’s disease (AD)-related risk gene (ATP-binding cassette A7-*ABCA7* and Clusterin-*CLU*) on the functional connectivity pattern of default mode network (DMN) in healthy middle-age adults.

**Methods:**

A total of 147 healthy middle-aged volunteers were enrolled in this study. All subjects completed MRI scans, neuropsychological assessments, and AD-related genotyped analysis. All subjects were divided into high, middle and low risk groups according to the score of risk genotypes, which included *CLU* (*rs11136000*, *rs2279590*, *rs9331888*, and *rs9331949*) and *ABCA7* (*rs3764650* and *rs4147929*). The genetic effects of CLU, ABCA7, and CLU × ABCA7 on DMN functional connectivity pattern were further explored. Moreover, the genetic effect of Apolipoprotein ε4 (APOEε4) was also considered. Finally, correlation analysis was performed between the signals of brain regions with genetic effect and neuropsychological test scores.

**Results:**

Compared with the low-risk group, the high-risk group of *CLU* showed decreased functional connectivity in posterior cingulate cortex (PCC) and the left middle frontal cortex (*P* < 0.05, GRF correction). As for the interaction between the *CLU* and *ABCA7*, all the subjects were divided into high, middle, and low risk group; the middle-risk group was divided into *CLU* and *ABCA7*-dominated middle-risk group. The function connectivity pattern of DMN among the three or four groups were distributed in the bilateral medial prefrontal cortex (MPFC) and bilateral superior frontal gyrus (SFG) (*P* < 0.05, GRF correction). When APOEε4 carriers were excluded, the *CLU*-predominant middle-risk group displayed the decreased functional connectivity in MPFC when compared with the low-risk group, while *ABCA7*-prodominant middle-risk group displayed decreased functional connectivity in cuneus when compared with the high-risk group (all *P* < 0.05, GRF correction). The *z* values of left middle frontal cortex were positively correlated with the scores of Serial Dotting Test (SDT) in high-risk group of *CLU*, while *z* values of MPFC and cuneus were positively correlated to the scores of Montreal Cognitive Assessment (MoCA) in low-risk group of three or four groups.

**Conclusion:**

The functional connectivity of MPFC-PCC might be modulated by the interaction of *CLU* and *ABCA7*. Moreover, *APOEε4* might be interacted with *ABCA7* and *CLU* modulation in the middle-aged carriers.

## Introduction

Alzheimer’s disease (AD) is a neurodegenerative disease with complex genetic characteristics (Lane et al., 2018). According to the findings of some genetic research, *apolipoprotein ε4* (*APOEε4*) has been considered as the major high risk genes for AD (Rajabli et al., 2018). *CLU* and *ABCA7* are listed as the top high risk genes secondary to *APOEε4* (Corneveaux et al., 2010; Nelson et al., 2017) and both have the similar roles in lipid metabolism, immune response and clearance of Aβ aggregation (Harold et al., 2009; Tan et al., 2016). It was found that the *CLU* and *ApoE* risk variants had combined effects on both volumetric expansion and lateral ventricle enlargement in the elderly (Yu et al., 2010; Roussotte et al., 2014). ABCA7 had been verified to be an independent predictor of AD (Liao et al., 2014). It is thus indeed important to study the combined effects of *CLU* and *ABCA7* on brain to explore the underlying common pathophysiologic mechanism of AD.

Neuroimaging has been widely applied to investigate the effects of AD-related genes on brain function and structure (Patel et al., 2013; Valkanova and Ebmeier, 2014). The default mode network (DMN) is one of the most vulnerable targets during the development of AD (Greicius et al., 2004). There was significant decline in functional connectivity among core regions of DMN in AD-related genes carriers with normal cognition. Prominent features of functional connectivity within DMN included (1) an overall strong level of interaction between the precuneus/posterior cingulate region and the rest of the DMN; (2) a high degree of interaction between the left and right medial temporal lobes (MTLs) combined with weak interactions between the MTLs and the rest of DMN; (3) strong interactions between the precuneus/posterior cingulate cortex (PCC) and the left inferior parietal lobe as well as between the dorsal and ventral sections of the medial prefrontal cortex (MPFC) (Fransson and Marrelec, 2008; Patel et al., 2013). Ye et al. found that *CLU* may affect the functional connectivity in the prefrontal cortex of AD patients, and *CLU-T* allele might be associated with compensatory neurological processes in the alteration of DMN in elderly subjects (Ye et al., 2017). Sinha et al. (2018) found that ABCA7 genetic risk differentially affects intra- MTL functional connectivity between MTL subfields, versus internetwork connectivity of the MTL with the MPFC, in non-demented older African Americans. Liao et al. (2014) found that *ABCA7 rs3764650* was significantly associated with AD and the influence of *ABCA7* was only evident in individuals without *APOEε4* alleles in elderly subjects (Liao et al., 2014). Mcfall et al. (2016) found that the cumulative genetic risk of *APOE* plus *CLU* had relation to executive function performance in older adults. Considering the findings of the above studies, we speculated that CLU and ABCA7 may have a early regulation of DMN, and ABCA7 and CLU and their genetic polymorphisms could affect DMN functional connectivity.

Thus, the purpose of this study is to investigate the impact of ABCA7 and CLU on the functional connectivity pattern of DMN in healthy middle-age adults and further explore the possible pathogenic mechanism of AD.

## Materials and Methods

### Subjects

This study enrolled 147 middle age adults with normal cognition from the local community between May 2015 and January 2017. All subjects were 45–64 years old (Fennemanotestine et al., 2011; Natalya et al., 2013), right-handedness. All the participants underwent MRI scans, neuropsychological tests and genotype analysis. The exclusion criteria were as follow: (1) obvious systematic diseases; (2) history of psychiatric or neurological diseases; (3) previous head trauma history; (4) drug or alcohol abuse; (5) MR imaging contraindications; (6) head motions with translation more than 1.0 mm or rotation more than 1.0°. This study was approved by the local Ethics Committee and all subjects provided written informed consent.

### Neuropsychological Assessments

All volunteers completed the following neuropsychological assessments before MR examination. Mini-Mental State Examination (MMSE) and Montreal Cognitive Assessment (MoCA) tests were used to measured global cognitive states, and MMSE > 26 score and MoCA > 26 score are identified as having intact cognition (Galea and Woodward, 2005; Nasreddine et al., 2005). Number Connection Test type A (NCT-A) and Digit Symbol Test (DST) were used to assess attention/executive function. Self-rating Anxiety Scale (SAS) and Self-rating Depression Scale (SDS) were used to assess the anxiety and depression state. Other neuropsychological assessments such as Line Tracing Test (LTT) and Serial Dotting Test (SDT) were used to assess executive function, attention or information processing speed (Salmon and Lange, 2001; Sankari et al., 2011).

### Genotyping and Grouping

We collected subjects between May 2015 and January 2017, all volunteer, none of the volunteers met the exclusion criteria. All the participants underwent MRI scans, neuropsychological tests. Each qualified volunteer draws 2 ml fasting blood before or after examination. All the samples were collected and stored in the −80°C after ED-TA anticoagulation, and were sent to Shanghai Tianhao Biotechnology company for genomic DNA extraction and SNP testing. All subjects were genotyped for *CLU* and *ABCA7* mutations. SNPs of *CLU* and *ABCA7*, including *CLUrs11136000*, *CLUrs2279590*, *CLUrs9331888*, *CLUrs9331949*, *ABCA7rs3764650*, and *ABCA7rs4147929* were reported to have high correlation with the onset of the late onset AD (Shi et al., 2012; Karch and Goate, 2015). Specifically, *CLUrs11136000-T*, *rs2279590-T*, *rs9331888-G* and *rs9331949-C* and *ABCA7rs3764650-G*, *rs4147929-A* are the risk alleles (Supplementary Table S1). Subsequently, each subject was recorded as 2, 1, or 0 score according to the amount of risk allele (Chouraki et al., 2016). There were 13 subjects with *APOE* ε4. APOE ε4 (*n* = 13) has been considered as the main risk gene of AD (Abushakra et al., 2017), and previous studies have shown the altered functional or structural connectivity pattern of DMN in *APOEε4* healthy carriers (Filippini et al., 2009; Machulda et al., 2011). Firstly, we excluded the subjects with *APOEε4*. Based on the distribution of scores of risk alleles of *CLU* and *ABCA7* without *APOE* ε4 (n = 13), the subjects were divided into the *CLU* low-risk group (0, 1, and 2 scores, *n* = 93) and *CLU* high risk group (3, 4, 5, and 6 scores, *n* = 41). Similarly, *ABCA7* (0 and 1 scores, *n* = 70) as low-risk group, *ABCA7* (2, 3, 4, 5, and 6 scores, *n* = 64) as high-risk group (Sleegers et al., 2015). Then, in all subjects included *APOEε4*. In order to explore the interactive effect of *CLU* and *ABCA7*, all subjects were further divided into high-risk (GG + GG, GG + GT, GC + GG, and GC + GT, *n* = 56), middle-risk (GG + TT, GC + TT, CC + GG, and CC + GT, *n* = 63), and low-risk group (CC + TT, *n* = 28) according to *CLU rs933188* and *ABCA7rs3764650*. Furthermore, we divided middle-risk group into *CLU*-dominated (GG + TT and GC + TT, *n* = 22) and ABCA7-dominated (CC + GG and CC + GT, *n* = 41). Considering that *APOEε4* might have an impact on the subjects. We further analyzed the interactive effect in *APOEε4* non-carriers. Similarly, the genotypes of the *APOE, ε2*, *ε3*, and *ε4* were determined by testing the SNPs *rs429358* and *rs7412* (Rajan et al., 2019) (Supplementary Table S2).

### MR Imaging Data Acquisition

All subjects were scanned on a 3.0-T MR scanner (TIM Trio, Siemens, Germany) which equipped with the standard 12-channel head coil. Foam paddings were used to minimize head motion. During the MRI scans, all participants were instructed to rest with their eyes closed and avoid thinking anything particular. T2 fluid attenuated inversion-recovery (T2-FLAIR) images were obtained to exclude the silent injury in brain, with its parameters as following: 25 axial slices, slice thickness = 4 mm, slice gap = 1.2 mm, image matrix = 232 × 256, field of view [FOV] = 220 × 220 mm^2^, repetition time [TR]/echo time [TE] = 9000/93 ms, flip angle = 130°, and inversion time = 2,500 ms. To obtain rs-fMRI data, we used a single-shot, gradient-recalled echo planar imaging sequence aligned along the anterior-posterior commissure to cover the whole brain which lasting 500 s. The parameters were as following: 250 volumes, image matrix = 64 × 64, FOV = 240 × 240 mm^2^, voxel size = 3.75 × 3.75 × 4 mm^3^, 30 axial slices, TR/TE = 2,000/40 ms, flip angle = 90°. Besides, a magnetization-prepared rapid gradient-echo sequence was conducted for acquiring the high-resolution, T1-weighted 3D anatomical images (T1W-3D-MPRAGE) with the parameters as follows: TR/TE = 2300/2.98 ms, flip angle = 9°, 176 slices, FOV = 256 × 256 mm^2^, acquisition matrix = 256 × 256, slice thickness = 1 mm (Wang et al., 2017; Kura et al., 2018).

### Data Preprocessing

All the image preprocessing was conducted using the Statistical Parametric Mapping software^1^ (SPM) based on MATLAB platform. Considering the subjects’ adaptation to the scanning state, first 10 volumes of each fMRI scan were discarded for the signal equilibrium. Then, the remaining 240 time points were processed (Andrewshanna et al., 2010). We excluded the subjects with translation or rotation parameters more than 1.0 mm or 1.0 degree (39 subjects were excluded due to movement). The T1W-3D-MPRAGE structure images were co-registered to the functional MRI data. Segmentation of T1WI was conducted into gray matter, white matter, and cerebrospinal fluid, and T1WI was spatially normalized to the Montreal Neurological Institute space with a voxel size of 3 × 3 × 3 mm^3^ by using unified segmentation algorithm, and smoothed by convolution with an isotropic Gaussian kernel of 6 mm. Finally, the processed data were detrended and nuisance covariates were regressed as well.

### Functional Connectivity Analysis

Default mode network was constructed by the method of PCC-based functional connectivity (Lee and Xue, 2018). PCC was determined by the sphere [radius with 8 mm, centered by MNI coordinates (−5, −49, and 40)] (Fernándezespejo et al., 2010). The average time series based on the seed was extracted, and the positive value of function connectivity of each voxel in the whole brain was calculated and transformed to *z* values by Fisher transform.

### Statistical Analysis

The demographic or neuropsychiatric data were analyzed using the SPSS version 20.0 (SPSS Inc., Chicago, IL, United States). The Kolmogorov-Smirnov test was conducted to assess the normality of quantitative data. Distributed data were expressed as mean ± standard deviation and then assessed by two sample *t*-test and ANOVA test. Independent sample non-parametric testing was used to analyze the data with non-normal distribution, which were reported as median and inter-quartile range [M (QU − QL)].

ANCOVA and two-sample *t*-tests were performed to detect the functional connectivity differences of DMN among the groups with different risk alleles. Age, gender, and education were set as covariates. All results were then corrected by the Gaussian random field (GRF) (*P* < 0.001 at the voxel level and *P* < 0.05 at the cluster level). *z* values of brain regions with statistical differences were extracted and correlated with the scores of the neuropsychological tests across all subjects as well as in each group, respectively. Correlation analyses were performed by using Pearson (normal distribution data) or Spearman correlation analysis (non-normal distribution data). *P* < 0.05 was regarded as significantly different.

## Results

### Demographical and Neuropsychological Results

This study included 147 healthy middle-aged (with 13 APOEε4 carriers). Mostly of our subjects were female (45/89 of Male/Female without APOE and 49/98 with APOE, average age of female with APOE is 55, and average age of female without APOE is 55). By calculation of the risk genotypes of *CLU* (*rs11136000*, *rs2279590*, *rs9331888*, and *rs9331949*) and *ABCA7* (*rs3764650* and *rs4147929*), there were no significant differences for age, sex, educational level or neuropsychological tests between high-risk and low-risk groups, respectively, in *CLU* and *ABCA7* (all *P* > 0.05) (Supplementary Tables S3, S4). Besides, there were no significant differences for age, sex, educational level or neuropsychological tests among the three groups (low-risk group, middle-risk group, and high-risk group) and the four groups (low-risk group, *CLU*-predominant middle-risk group, *ABCA7*-prodominant middle-risk group, and high-risk group) (all *P* > 0.05) (Supplementary Tables S5–S8).

### Function Connectivity of DMN Altered by the Risk Degree of *CLU* and *ABCA7*

**FIGURE 1:**
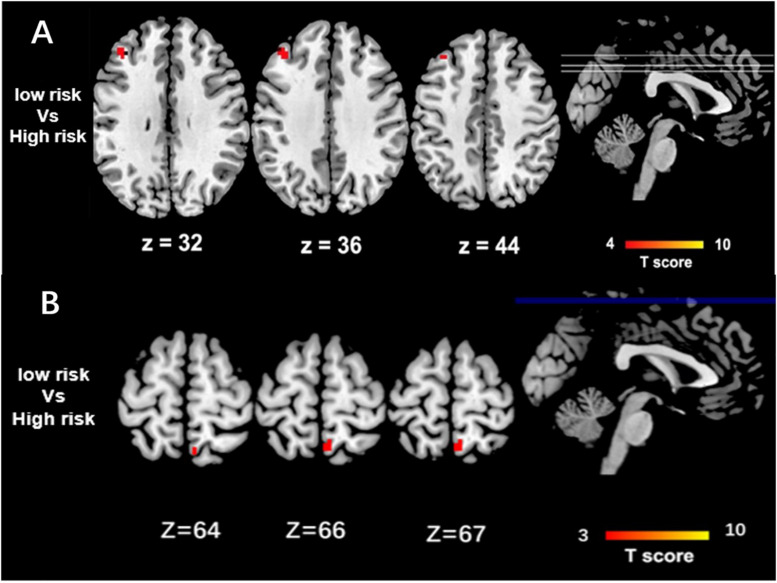
Group Differences of Functional Connectivity in CLU and ABCA7. **(A)** The two-sample *t*-test between risk degree of the total risk gene scores in CLU and DMN shows that the major differences between high-risk and low-risk group are located in the left middle frontal cortex (*P* < 0.05 after GRF correction). Based on the distribution of scores of risk alleles of *CLU* without *APOEε4*. High-risk = scored 3, 4, 5, and 6; low-risk = scored 0, 1, and 2. **(B)** The two-sample *t*-test between risk degree of the total risk gene scores in ABCA7 and DMN shows that the major differences between high-risk and low-risk group are located in the right precuneus, while there is no significant difference between the two groups (*P* < 0.05 after GRF correction). Based on the distribution of scores of risk alleles of *ABCA7* without *APOEε4*. High-risk = GG + GG/GG + GT/GC + GG/GC + GT; low-risk = CC + TT.

High-risk *CLU* group showed decreased function connectivity in PCC and the left middle frontal cortex, when compared with the low-risk carriers (*P* < 0.05 after GRF-corrected) (Figure 1A). Functional connectivity pattern of DMN modulated by the risk of ABCA7 was distributed in the right precuneus, while no significant difference was found after GRF correction (Figure 1B).

### Function Connectivity of DMN Influenced by the Interaction of *CLU* and *ABCA7*

**FIGURE 2:**
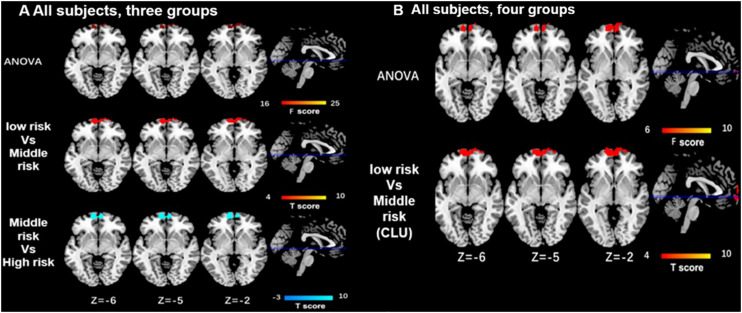
Differences of functional connectivity influenced by the interaction of CLU and ABCA7 in all subjects. **(A)** In all subjects, the major differences of function connectivity of DMN among the three groups are distributed in the bilateral medial prefrontal cortex (MPFC) and bilateral superior frontal gyri (SFG). When compared with the low- and high-risk group, middle-risk group displays the lower functional connectivity in MPFC/SFG (i < 0.05 after GRF correction). **(B)** In all subjects, the differences resulted from ANOVA test among four groups are located in the bilateral MPFC, while the low-risk group displays the increased functional connectivity in MPFC only when compared with CLU-predominant middle-risk group (*P* < 0.05 after GRF correction). All subjects included *APOEε4*. interactive effect of *CLU* and *ABCA7*, high-risk = GG + GG/GG + GT/GC + GG/GC + GT; middle-risk = GG + TT/GC + TT/CC + GG/CC + GT; low-risk = CC + TT; middle-risk1 (*CLU*-dominated) = GG + TT/GC + TT; middle-risk2 (ABCA7-dominated) = CC + GG/CC + GT.

**FIGURE 3:**
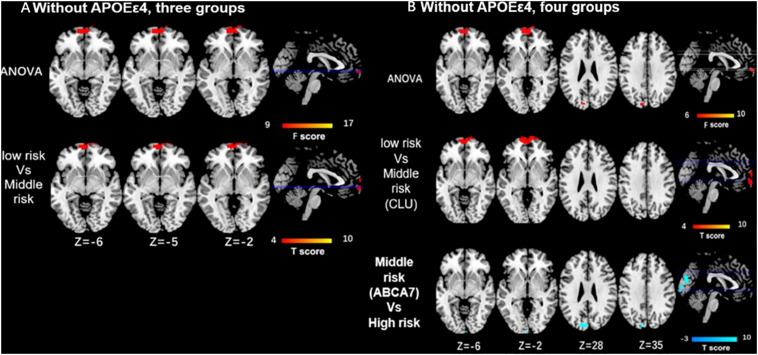
Differences of functional connectivity influenced by the interaction of CLU and ABCA7 in subjects without APOEε4. **(A)** Without including APOEε4, the major differences among the three groups are located in the MPFC/SFG, and the low-risk group displays the enhanced activity in MPFC/SFG when compared with middle-risk group (*P* < 0.05 after GRF correction). **(B)** ANOVA resulted from four groups demonstrates the altered activity in MPFC and cuneus. Specifically, the low-risk group shows enhanced functional connectivity in MPFC when compared with CLU-predominant middle-risk group, while the high-risk group displays increased activity in cuneus when compared with ABCA7-prodominant middle-risk group (*P* < 0.05 after GRF correction). All subjects without *APOEε4*. Interactive effect of *CLU* and *ABCA7*, high-risk = GG + GG/GG + GT/GC + GG/GC + GT; middle-risk = GG + TT/GC + TT/CC + GG/CC+GT; low-risk = CC + TT; middle-risk1 (*CLU*-dominated) = GG + TT/GC + TT; middle-risk2 (ABCA7-dominated) = CC + GG/CC + GT.

As for the interaction between the *CLU rs9331888* and *ABCA7 rs376465*, function connectivity pattern of DMN among the three groups or the four groups were distributed in the bilateral MPFC and bilateral superior frontal gyrus (SFG). The *CLU*-predominant middle-risk group displayed the decreased functional connectivity in MPFC when compared with the low risk group (*P* < 0.05 after GRF-corrected), while ABCA7-prodominant middle-risk group displayed decreased activity in cuneus when compared with the high-risk group (*P* < 0.05 after GRF-corrected). When *APOEε4* carriers were excluded, the ANOVA test result among four groups demonstrated the altered activity in MPFC and cuneus. Specifically, the *CLU*-predominant middle-risk group displayed the decreased functional connectivity in MPFC when compared with the low-risk group, while *ABCA7*-prodominant middle-risk group displayed decreased activity in cuneus when compared with the high-risk group (*P* < 0.05 after GRF-corrected) (Table 1 and Figures 2, 3).

**TABLE 1:** Differences of functional connectivity among different groups.

**Groups**	**Region (AAL)**	**Cluster size**	**MNI coordinates (mm)**			**Peak *t* value**
			***x***	***y***	***z***	
**Low risk, middle risk, and high risk (all subjects)**						
ANOVA	MPFC/SFG (L/R)	161	18	72	9	16.729
Low risk vs middle risk	MPFC/SFG (L/R)	250	18	72	9	4.090
Middle risk vs high risk	MPFC/SFG (L/R)	183	−9	57	−18	–3.835
**Low risk, middle risk (CLU), middle risk (ABCA7), and high risk (all subjects)**						
ANOVA	MPFC/SFG (L/R)	198	18	72	9	6.619
Low risk vs middle risk(CLU)	MPFC (L/R)	323	18	72	9	4.23
**Low risk, middle risk and high risk (without APOEε4)**						
** ANOVA**	MPFC (L/R)	147	21	72	6	9.148
Low risk vs middle risk	MPFC/SFG (L/R)	196	18	72	6	4.174
**Low risk, middle risk (CLU), middle risk (ABCA7), and high risk (without APOEε4)**						
ANOVA	MPFC/SFG (L/R)	138	21	72	6	6.286
Low risk vs middle risk (CLU)	MPFC/SFG (L/R)	295	18	72	6	4.107
Middle risk vs high risk (ABCA7)	CUN (L)	181	−12	−84	30	–3.225

*With GRF corrected (*P* < 0.001 at the voxel level and *P* < 0.05 at the cluster level).*

### Correlation Analysis Results

The *z* values of left middle frontal cortex extracted from the result of ANOVA were positively related to the scores of SDT in high-risk group of CLU (*r* = 0.335, *P* = 0.037). Furthermore, based on genotyping of *CLU rs9331888* and *ABCA7 rs3764650*, the *z* values of MPFC and cuneus distracted from the result of ANOVA were positively related to the scores of MoCA in low-risk group of among the three groups or the four groups (*P* < 0.05) (Supplementary Tables S9, S10).

## Discussion

This study demonstrated that MPFC-PCC was the common target brain region modulated by the interaction of *CLU* and *ABCA7*. Moreover, *APOEε4* might be interacted with *ABCA7* and *CLU* modulation in the middle-aged carriers.

Our research indicated that *CLU-G* and *ABCA7-G* could impact DMN connectivity, and MPFC-PCC was the common target modulated by the interaction of *CLU* and *ABCA7*. For ABCA7, ANOVA test results resulted from four groups demonstrated the altered activity in cuneus. However, there was no significant difference between the two groups after GRF correction, indicating that the effect on the functional connectivity of DMN may be weak. Zhang et al. (2015) studied the effects of *PICALM* and *CLU* on resting-state functional connectivity of hippocampus in healthy young people and found the interaction between two genotypes. Liao et al. (2014) found that there was a certain correlation between *ABCA7* and *APOEε4* in Han population. While a study of Han people with AD in northern China confirmed that *ABCA7 rs3764650* was associated with AD (Liu et al., 2014; Choi et al., 2017). In our study, the *CLU*-predominant middle-risk group displayed the decreased functional connectivity in MPFC when compared with the low risk group, indicating that *CLU-G* and *ABCA7-G* could impact DMN connectivity. MPFC is thought to be related to cognitive and can dynamically regulate processing of emotion. It is closely related to the function of the limbic system and can integrate information from the internal and external environment and emotional information (Fransson and Marrelec, 2008; Kwapis et al., 2015). Many studies suggested that there was a difference of DMN in patients with AD in MPFC which was similar to our results (Beason-held, 2011; Wang et al., 2017; Kura et al., 2018). Tan et al. (2016) and Ye et al. (2017) found that *CLU* could continue affect the functional connectivity of DMN in the MPFC, indicating that MPFC-PCC was the common target modulated by the interaction of *CLU* and *ABCA7*.

*APOEε4* is a recognized risk gene for AD (Andrewshanna et al., 2010). *APOEε4* might have an influence on the effect of *CLU* and *ABCA7*. In our study, when *APOEε4* carriers were excluded, the *CLU*-predominant middle-risk group displayed the decreased functional connectivity in MPFC when compared with the low-risk group, while *ABCA7*-prodominant middle-risk group displayed decreased activity in cuneus when compared with the high-risk group. DeMattos et al. (2004) pointed out that *APOEε4* and *CLU* could cooperate to inhibit the deposition of Aβ, *APOEε4* and *CLU* might severely alter the clearance of Aβ on the blood-brain barrier (Haight et al., 2018). In another study of brain functional connectivity in healthy young adults, the DMN functional connectivity of *APOEε4* carriers was enhanced (Nagai et al., 2004). Consistent with our results that when *APOEε4* carriers were excluded, the ANOVA resulting from four groups demonstrated the altered activity in MPFC and cuneus, which indicated that *APOEε4* might be interacted with *ABCA7* and *CLU* modulation in the middle-aged carriers. Otherwise, the changes of functional connectivity density were significantly correlated with neuropsychological scores. Studies have shown the short-term functional connectivity density decreases in the PCC and precuneus, increases in the medial frontal cortex and middle frontal gyrus (Zhao et al., 2017). This is consistent with the results of our study that the functional connectivity changes of MPFC are significantly correlated with the scores of MoCA.

This study had some limitations. Firstly, we only chose middle age adults for investigating the influence of *ABCA7* and *CLU* on the DMN function connectivity. Natalya et al. (2013) found that the influence of *CLU* genotype was found to be higher in the subjects older than 50 years old. Fennemanotestine et al. (2011) found that presence of *APOEε4* was associated with thinner frontal cortex in middle age adults. However, all the subjects were middle-aged, and the differences in image representation might be small, which could not fully explain the differences in gene effects. Secondly, *ε2* genotype was reported as a protective gene for delayed AD, the influence of *ε2* was not further observed in this study. Thirdly, other factors, including social, economic, family composition and some other conditions should be taken into account in the future to exclude the impact of environmental factors.

In summary, this study indicates MPFC-PCC connectivity was the common target which was modulated by the interaction of *CLU* and *ABCA7*. Moreover, *APOEε4* might be interacted with *ABCA7* and *CLU* modulation in the middle-aged carriers.

## Data Availability Statement

The original contributions presented in the study are included in the article/Supplementary Material, further inquiries can be directed to the corresponding author.

## Ethics Statement

The studies involving human participants were reviewed and approved by Affiliated Jinling Hospital, Medical School of Nanjing University. The patients/participants provided their written informed consent to participate in this study.

## Author Contributions

XZ analyzed data and wrote the first draft of the manuscript. YW and LiZ contributed equally to this work in study design, literature search, and manuscript editing. HZ and LL were responsible for recruiting the subjects and collecting data. GL reviewed and revised the manuscript. LoZ designed the study, conducted the initial analyses, and reviewed and revised the manuscript. All authors have contributed to and have approved the final manuscript.

## Conflict of Interest

The authors declare that the research was conducted in the absence of any commercial or financial relationships that could be construed as a potential conflict of interest.

## Footnotes

1 http://www.fil.ion.ucl.ac.uk/spm

## Supplementary Material

TABLE S1 CLU rs11136000, rs2279590, rs9331888, rs9331949 score, and ABCA7 rs3764650, rs4147929 score.

TABLE S2 Genotypes of CLUrs9331888 and ABCA7rs3764650.

TABLE S3 Demographic, clinical, and neuropsychological data of CLU risk gene scores without APOE.

TABLE S4 Demographic, clinical, and neuropsychological data of ABCA7 risk gene scores without APOE.

TABLE S5 Demographic, clinical, and neuropsychological data of three groups (low-risk group, middle-risk group, high-risk group) with APOE.

TABLE S6 Demographic, clinical, and neuropsychological data of four groups (low-risk group, CLU-predominant middle-risk group, ABCA7-prodominant middle-risk group, high-risk group) with APOE.

TABLE S7 Demographic, clinical, and neuropsychological data of three groups (low-risk group, middle-risk group, high-risk group) without APOE.

TABLE S8 Demographic, clinical, and neuropsychological data of four groups (low-risk group, CLU-predominant middle-risk group, ABCA7-prodominant middle-risk group, high-risk group) without APOE.

TABLE S9 Correlation analysis results of the z values of MPFC and cuneus distracted from the result of ANOVA with APOE.

TABLE S10 Correlation analysis results of the z values of MPFC and cuneus distracted from the result of ANOVA without APOE.

## Abbreviations

ABCA7: ATP-binding cassette A7
AD: Alzheimer’s disease
APOE: apolipoprotein E
CLU: clusterin
CUN: cuneus
DMN: default mode network
MFG: middle frontal gyrus
MPFC: medial prefrontal cortex
PCC/PCu: posterior cingulate cortex/precuneus
rs-fMRI: resting-state functional MR imaging
SFG: superior frontal gyrus.
