# Supplementary material for: Impacts of AD-Related ABCA7 and CLU Variants on Default Mode Network Connectivity in Healthy Middle-Age Adults
Source: Front Mol Neurosci. 2020 Jul 31;13:145. doi: 10.3389/fnmol.2020.00145 (PMC7412986; doi:10.3389/fnmol.2020.00145)
Supplement: TABLE S1 — CLU rs11136000, rs2279590, rs9331888, rs9331949 score, and ABCA7 rs3764650, rs4147929 score. [file Table_1.DOCX]

**Supplementary Materials**

**Supplementary Table 1. CLU rs11136000, rs2279590, rs9331888, rs9331949 score and ABCA7 rs3764650, rs4147929 score**

| CLU | rs11136000 | CC 0 score  CT 1 score  TT 2 score |
| --- | --- | --- |
|  | rs2279590 | CC 0 score  CT 1 score  TT 2 score |
|  | rs9331888 | CC 0 score  GC 1 score  GG 2 score |
|  | rs9331949 | TT 0 score  CT 1 score  CC 2 score |
| ABCA7 | rs3764650 | TT 0 score  GT 1 score  GG 2 score |
|  | rs4147929 | GG 0 score  GA 1 score  AA 2 score |

The score of CLU and ABCA7. SNPs of *CLU* and *ABCA7*, including *CLUrs11136000, CLUrs2279590, CLUrs9331888, CLUrs9331949*, *ABCA7rs3764650* and *ABCA7rs4147929. CLUrs11136000-T, rs2279590-T, rs9331888-G* and *rs9331949-C* and *ABCA7 rs3764650-G, rs4147929-A* are the risk alleles. Each subject is recorded as 2, 1 or 0 score according to the amount of risk allele.

**Supplementary Table 2：Genotypes of CLUrs9331888 and ABCA7rs3764650**

| Low risk | CC+TT | |
| --- | --- | --- |
| Mid risk | GG+TT  GC+TT  CC+GG  CC+GT | CLU(mid1)  GG+TT/GC+TT |
|  |  | ABCA7 (mid2)  CC+GG/ CC+GT |
| High risk | GG+GG  GG+GT  GC+GG  GC+GT | |

According to *CLU rs933188* and *ABCA7rs3764650,* all subjects were divided into high-risk (GG+GG, GG+GT, GC+GG, GC+GT), middle-risk (GG+TT, GC+TT, CC+GG, CC+GT) and low-risk group (CC+TT). Furthermore, we divided middle-risk group into CLU-dominated (GG+TT, GC+TT) and ABCA7-dominated (CC+GG, CC+GT).

**Supplementary Table 3：Demographic, clinical, and neuropsychological data of CLU risk gene scores without APOE**

| **Variables** | **Low risk (n=93)** | **High risk (n=41)** | **P value** |
| --- | --- | --- | --- |
| Age (y) | 54.9±5.0 | 54.9±4.7 | 0.801^b^ |
| Gender(M/F) | 32/61 | 13/28 | 0.264^b^ |
| EDU(y) | 8.9±3.4 | 9(12-6) | 0.423^a^ |
| MMSE/30 | 29(30-28) | 28.5±1.4 | 0.812^a^ |
| MOCA/30 | 25.4±3.1 | 25.9±2.6 | 0.466^b^ |
| NCT-A (s) | 55(70-42) | 55.8±17.6 | 0.636^a^ |
| DST(n) | 36.1±13.1 | 38.0±13.6 | 0.491^b^ |
| LTT(s) | 63.7±27.3 | 77.3±41.9 | 0.310^b^ |
| SDT(s) | 46.6±14.0 | 48.8±15.9 | 0.436^b^ |
| SAS (score) | 29.5±7.9 | 27.4±5.2 | 0.526^b^ |
| SDS (score) | 29.6±9.3 | 28±6.3 | 0.291^b^ |

Mean ± standard deviation, median and inter-quartile range [M (QU-QL)]

S = second, n = number, y = year, EDU = education, MMSE = Mini-Mental State Examination, MoCA = Montreal Cognitive Assessment, NCT-A = number connection test type A, DST = digit symbol test, LTT = Line tracing test, SDT = serial dotting test, SAS = Self-rating Anxiety Scale, SDS = Self-rating Depression Scale.

a The k-independent sample nonparametric tests

b Analysis of variance

**Supplementary Table 4：Demographic, clinical, and neuropsychological data of ABCA7 risk gene scores without APOE**

| **Variables** | **Low risk (n=70)** | **High risk (n=64)** | **P value** |
| --- | --- | --- | --- |
| Age (y) | 55.8±4.5 | 54.0±5.0 | 0.482^b^ |
| Gender(M/F) | 23/47 | 22/42 | 0.054^b^ |
| EDU(y) | 9(12-9) | 9(10.5-6) | 0.303^a^ |
| MMSE/30 | 29(29-27.2) | 29(30-28) | 0.341^a^ |
| MOCA/30 | 26(27-24) | 26.0±2.6 | 0.151^a^ |
| NCT-A (s) | 63.2±22.1 | 54.8±20.7 | 0.600^b^ |
| DST(n) | 34.6±12.3 | 38.5±14.0 | 0.235^b^ |
| LTT(s) | 72.3±30.27 | 58(78.5-45) | 0.084^a^ |
| SDT(s) | 51.8±14.3 | 43.1±13.7 | 0.279^b^ |
| SAS (score) | 29.2±7.2 | 28.8±7.5 | 0.454^b^ |
| SDS (score) | 27(32-22) | 29.2±8.6 | 0.756^a^ |

Mean ± standard deviation, median and inter-quartile range [M (QU-QL)]

s=second, n=number, y=year, EDU = education, MMSE = Mini-Mental State Examination, MoCA = Montreal Cognitive Assessment, NCT-A = number connection test type A, DST = digit symbol test, LTT = Line tracing test, SDT = serial dotting test, SAS = Self-rating Anxiety Scale, SDS = Self-rating Depression Scale.

a The k-independent sample nonparametric tests

b Analysis of variance

**Supplementary Table 5：Demographic, clinical, and neuropsychological data of three groups (low-risk group, middle-risk group, high-risk group) with APOE**

| **Variables** | **Low risk (n=28)** | **middle risk (n=63)** | **High risk (n=56)** | **P value** |
| --- | --- | --- | --- | --- |
| Age (y) | 56.0±4.4 | 54.7±4.7 | 54.9±5.3 | 0.49^b^ |
| Gender(M/F) | 9/19 | 22/41 | 18/38 | 0.186^b^ |
| EDU(y) | 9.4±3.8 | 9.0(9.0-6.0) | 9.0(12.0-6.0) | 0.517^a^ |
| MMSE/30 | 29.0(30.0-27.0) | 29.0(29.0-28.0) | 29.0(30.0-28.0) | 0.91^b^ |
| MOCA/30 | 24.4±3.5 | 25.8±2.5 | 27.0(28.0-24.0) | 0.134^a^ |
| NCT-A (s) | 61.8±19.8 | 61.2±28.7 | 55.0±20.8 | 0.317^b^ |
| DST(n) | 34.5±10.1 | 35.3±13.2 | 39.2±14.4 | 0.191^b^ |
| LTT(s) | 65.5±24.7 | 72.2±37.0 | 61.0(80.0-46.0) | 0.834^a^ |
| SDT(s) | 51.3±16.7 | 47.9±13.7 | 45.3±15.9 | 0.249^b^ |
| SAS (score) | 31.4±8.1 | 28.8±9.2 | 28.0(33.0-25.0) | 0.125^a^ |
| SDS (score) | 30.6±9.7 | 27.0(32.0-23.0) | 24.0(33.0-21.0) | 0.436^a^ |

Mean ± standard deviation, median and inter-quartile range [M (QU-QL)];

S = second, n = number, y = year, EDU = education, MMSE = Mini-Mental State Examination, MoCA = Montreal Cognitive Assessment, NCT-A = number connection test type A, DST = digit symbol test, LTT = Line tracing test, SDT = serial dotting test, SAS = Self-rating Anxiety Scale, SDS = Self-rating Depression Scale.

a The k-independent sample nonparametric tests; b Analysis of variance

**Supplementary Table 6：Demographic, clinical, and neuropsychological data of four groups (low-risk group, CLU-predominant middle-risk group, ABCA7-prodominant middle-risk group, high-risk group) with APOE**

| **Variables** | **Low risk (n=28)** | **Middle risk(CLU, n=22)** | **Middle risk(ABCA7, n=41)** | **High risk (n=56)** | **P value** |
| --- | --- | --- | --- | --- | --- |
| Age (y) | 56.0±4.4 | 52.3±4.3 | 55.9±4.5 | 54.9±5.3 | 0.055^b^ |
| Gender(M/F) | 9/19 | 8/14 | 12/29 | 18/38 | 0.475^b^ |
| EDU(y) | 9.4±3.8 | 9.0(9.7-6.0) | 9.0(9.0-7.5) | 9.0(12.0-6.0) | 0.442^a^ |
| MMSE/30 | 29.0(30.0-27.0) | 28.2±1.0 | 29.0(29.0-28.0) | 29.0(30.0-28.0) | 0.708^a^ |
| MOCA/30 | 24.4±3.5 | 25.9±2.6 | 25.8±2.5 | 27.0(28.0-24.0) | 0.242^a^ |
| NCT-A (s) | 61.8±19.8 | 54.8±21.9 | 64.7±31.5 | 55.0±20.8 | 0.183^b^ |
| DST(n) | 34.5±10.1 | 34.9±12.0 | 35.5±13.9 | 39.2±14.4 | 0.354^b^ |
| LTT(s) | 65.5±24.7 | 69.0±45.3 | 73.5±32.4 | 61.0(80.0-46.0) | 0.579^a^ |
| SDT(s) | 51.3±16.7 | 45.3±14.9 | 49.2±13.1 | 45.3±15.9 | 0.294^b^ |
| SAS (score) | 31.4±8.1 | 28.0±12.0 | 29.2±7.3 | 28.0(33.0-25.0) | 0.08^a^ |
| SDS (score) | 30.6±9.7 | 28.5±6.3 | 27.0(33.0-23.0) | 24.0(33.0-21.0) | 0.825^a^ |

Mean ± standard deviation, median and inter-quartile range [M (QU-QL)];

S = second, n = number, y = year, EDU = education, MMSE = Mini-Mental State Examination, MoCA = Montreal Cognitive Assessment, NCT-A = number connection test type A, DST = digit symbol test, LTT = Line tracing test, SDT = serial dotting test, SAS = Self-rating Anxiety Scale, SDS = Self-rating Depression Scale.

a The k-independent sample nonparametric tests; b Analysis of variance

**Supplementary Table 7：Demographic, clinical, and neuropsychological data of three groups (low-risk group, middle-risk group, high-risk group) without APOE**

| **Variables** | **Low risk (n=25)** | **middle risk (n=58)** | **High risk (n=51)** | **P value** |
| --- | --- | --- | --- | --- |
| Age (y) | 56.0±4.3 | 54.5±4.6 | 54.8±5.3 | 0.452^b^ |
| Gender(M/F) | 8/17 | 21/37 | 16/35 | 0.319^b^ |
| EDU(y) | 9.2±3.8 | 9.0(9.0-8.5) | 9.0(12.0-6.0) | 0.632^a^ |
| MMSE/30 | 29.0(30.0-27.0) | 29.0(29.0-28.0) | 29.0(29.2-28.0) | 0.843^b^ |
| MOCA/30 | 24.2±3.6 | 25.8±2.6 | 26.5(28.0-24.0) | 0.147^a^ |
| NCT-A (s) | 63.4±20.3 | 62.5±29.2 | 55.0±20.3 | 0.218^b^ |
| DST(n) | 33.3±9.8 | 35.0±13.3 | 39.4±14.4 | 0.105^b^ |
| LTT(s) | 65.1±25.2 | 73.2±37.2 | 66.2±30.3 | 0.444^b^ |
| SDT(s) | 52.4±16.8 | 48.8±13.7 | 44.2±16.3 | 0.088^b^ |
| SAS (score) | 30.9±7.5 | 26.0(32.2-22.0) | 28.0(32.2-25.0) | 0.092^a^ |
| SDS (score) | 30.1±8.7 | 27.0(32.2-22.0) | 24.5(33.5-22.0) | 0.677^a^ |

Mean ± standard deviation, median and inter-quartile range [M (QU-QL)]; s second, n number, y year,

S = second, n = number, y = year, EDU = education, EDU = education, MMSE = Mini-Mental State Examination, MoCA = Montreal Cognitive Assessment, NCT-A = number connection test type A, DST = digit symbol test, LTT = Line tracing test, SDT = serial dotting test, SAS = Self-rating Anxiety Scale, SDS = Self-rating Depression Scale.

a The k-independent sample nonparametric tests; b Analysis of variance

**Supplementary Table 8：Demographic, clinical, and neuropsychological data of four groups (low-risk group, CLU-predominant middle-risk group, ABCA7-prodominant middle-risk group, high-risk group) without APOE**

| **Variables** | **Low risk (n=25)** | **Middle risk(CLU, n=21)** | **Middle risk(ABCA7, n=37)** | **High risk (n=51)** | **P value** | |  |
| --- | --- | --- | --- | --- | --- | --- | --- |
| Age (y) | 56.0±4.3 | 52.6±4.3 | 55.7±4.5 | 54.8±5.3 | | 0.13^b^ | |
| Gender(M/F) | 8/17 | 8/13 | 12/25 | 16/35 | | 0.371^b^ | |
| EDU(y) | 9.2±3.8 | 9.0(9.0-6.0) | 9.0(9.0-9.0) | 9.0(12.0-6.0) | | 0.747^a^ | |
| MMSE/30 | 29.0(30.0-27.0) | 28.7±1.0 | 28.4±1.1 | 29.0(29.2-28.0) | | 0.756^a^ | |
| MOCA/30 | 24.2±3.6 | 25.8±2.6 | 25.8±2.6 | 26.5(28.0-24.0) | | 0.279^a^ | |
| NCT-A (s) | 63.4±20.3 | 55.6±20.8 | 65.8±32.9 | 55.0±20.3 | | 0.172^b^ | |
| DST(n) | 33.3±9.8 | 33.±11.4 | 35.6±14.4 | 39.4±14.4 | | 0.204^b^ | |
| LTT(s) | 65.1±25.2 | 71.0±45.6 | 74.4±32.5 | 66.2±30.3 | | 0.623^b^ | |
| SDT(s) | 52.4±16.8 | 46.0±14.9 | 50.4±12.9 | 44.2±16.3 | | 0.115^b^ | |
| SAS (score) | 30.9±7.5 | 28.0±12.3 | 28.8±7.4 | 28.0(32.2-25.0) | | 0.098^a^ | |
| SDS (score) | 30.1±8.7 | 28.4±6.4 | 26.0(32.0-22.5) | 24.5(33.5-22.0) | | 0.842^a^ | |

Mean ± standard deviation, median and inter-quartile range [M (QU-QL)];

S = second, n = number, y = year, EDU = education, MMSE = Mini-Mental State Examination, MoCA = Montreal Cognitive Assessment, NCT-A = number connection test type A, DST = digit symbol test, LTT = Line tracing test, SDT = serial dotting test, SAS = Self-rating Anxiety Scale, SDS = Self-rating Depression Scale.

a The k-independent sample nonparametric tests; b Analysis of variance

**Supplementary Table 9: Correlation analysis results of the z values of MPFC and cuneus distracted from the result of ANOVA with APOE**

|  | **Low risk** | | **middle risk** | | **High risk** | | **Low risk** | | **Middle risk(CLU)** | | **Middle risk(ABCA7)** | | **High risk** | |
| --- | --- | --- | --- | --- | --- | --- | --- | --- | --- | --- | --- | --- | --- | --- |
| **Variables** | **R value** | **P value** | **R value** | **P value** | **R value** | **P value** | **R value** | **P value** | **R value** | **P value** | **R value** | **P value** | **R value** | **P value** |
| Age (y) | -0.171 | 0.383 | -0.049 | 0.703 | -0.059 | 0.664 | -0.147 | 0.456 | 0.025 | 0.013 | -0.028 | 0.861 | -0.006 | 0.962 |
| EDU(y) | -0.228 | 0.243 | -0.219 | 0.085 | 0.116 | 0.394 | -0.159 | 0.420 | -0.238 | 0.287 | -0.218 | 0.170 | 0.973 | 0.000 |
| MMSE/30 | -0.069 | 0.726 | -0.124 | 0.334 | 0.228 | 0.095 | -0.116 | 0.564 | 0.050 | 0.824 | -0.221 | 0.166 | 0.217 | 0.111 |
| MOCA/30 | **-0.453** | **0.018** | -0.113 | 0.380 | 0.103 | 0.455 | **-0.409** | **0.034** | 0.035 | 0.877 | -0.269 | 0.089 | 0.102 | 0.459 |
| NCT-A (s) | 0.136 | 0.497 | 0.160 | 0.210 | -0.103 | 0.454 | 0.113 | 0.575 | 0.055 | 0.808 | 0.266 | 0.093 | -0.095 | 0.491 |
| DST(n) | -0.100 | 0.620 | -0.253 | 0.046 | 0.194 | 0.155 | -0.091 | 0.653 | -0.250 | 0.262 | -0.286 | 0.070 | 0.173 | 0.206 |
| LTT(s) | -0.068 | 0.736 | -0.12 | 0.354 | -0.243 | 0.079 | -0.045 | 0.822 | -0.149 | 0.518 | -0.054 | 0.737 | -0.201 | 0.150 |
| SDT(s) | -0.231 | 0.247 | 0.136 | 0.293 | -0.041 | 0.770 | -0.200 | 0.316 | 0.113 | 0.625 | 0.218 | 0.171 | 0.005 | 0.972 |
| SAS (score) | -0.281 | 0.156 | 0.082 | 0.520 | 0.016 | 0.909 | -0.334 | 0.089 | -0.057 | 0.801 | 0.308 | 0.050 | -0.007 | 0.962 |
| SDS (score) | -0.191 | 0.341 | 0.109 | 0.393 | 0.031 | 0.823 | -0.24 | 0.228 | -0.187 | 0.405 | 0.231 | 0.146 | 0.116 | 0.393 |

S = second, n = number, y = year, EDU = education, MMSE = Mini-Mental State Examination, MoCA = Montreal Cognitive Assessment, NCT-A = number connection test type A, DST = digit symbol test, LTT = Line tracing test, SDT = serial dotting test, SAS = Self-rating Anxiety Scale, SDS = Self-rating Depression Scale.

**Supplementary Table 10: Correlation analysis results of the z values of MPFC and cuneus distracted from the result of ANOVA without APOE**

|  | **Low risk** | | **middle risk** | | **High risk** | | **Low risk** | | **Middle risk(CLU)** | | **Middle risk(ABCA7)** | | **High risk** | |
| --- | --- | --- | --- | --- | --- | --- | --- | --- | --- | --- | --- | --- | --- | --- |
| **Variables** | **R value** | **P value** | **R value** | **P value** | **R value** | **P value** | **R value** | **P value** | **R value** | **P value** | **R value** | **P value** | **R value** | **P value** |
| Age (y) | -0.137 | 0.515 | -0.054 | 0.687 | -0.060 | 0.673 | -0.133 | 0.525 | 0.000 | 0.999 | -0.021 | 0.901 | 0.003 | 0.984 |
| EDU(y) | -0.152 | 0.469 | -0.261 | 0.048 | 0.076 | 0.595 | -0.075 | 0.722 | -0.206 | 0.371 | -0.265 | 0.113 | 0.07 | 0.627 |
| MMSE/30 | -0.303 | 0.149 | -0.110 | 0.412 | 0.190 | 0.186 | -0.316 | 0.132 | 0.075 | 0.748 | -0.242 | 0.149 | 0.185 | 0.198 |
| MOCA/30 | **-0.504** | **0.012** | -0.105 | 0.432 | 0.022 | 0.881 | **-0.472** | **0.020** | 0.052 | 0.823 | -0.268 | 0.109 | 0.052 | 0.718 |
| NCT-A (s) | 0.132 | 0.537 | 0.145 | 0.277 | -0.092 | 0.527 | 0.100 | 0.643 | 0.023 | 0.922 | 0.258 | 0.123 | -0.075 | 0.603 |
| DST(n) | -0.104 | 0.628 | -0.245 | 0.064 | 0.160 | 0.268 | -0.111 | 0.604 | -0.234 | 0.307 | -0.279 | 0.094 | 0.131 | 0.364 |
| LTT(s) | -0.012 | 0.955 | -0.117 | 0.385 | -0.213 | 0.146 | -0.002 | 0.992 | -0.173 | 0.466 | -0.041 | 0.809 | -0.177 | 0.229 |
| SDT(s) | -0.184 | 0.391 | 0.111 | 0.413 | -0.040 | 0.787 | -0.154 | 0.472 | 0.096 | 0.688 | 0.204 | 0.225 | 0.009 | 0.949 |
| SAS (score) | -0.284 | 0.179 | 0.187 | 0.160 | -0.132 | 0.361 | -0.313 | 0.136 | -0.059 | 0.800 | 0.311 | 0.061 | -0.164 | 0.255 |
| SDS (score) | -0.309 | 0.142 | 0.156 | 0.244 | 0.049 | 0.734 | -0.336 | 0.108 | -0.178 | 0.44 | 0.279 | 0.095 | 0.013 | 0.927 |

S=second, n=number, y=year, EDU = education, MMSE = Mini-Mental State Examination, MoCA = Montreal Cognitive Assessment, NCT-A = number connection test type A, DST = digit symbol test, LTT = Line tracing test, SDT = serial dotting test, SAS = Self-rating Anxiety Scale, SDS = Self-rating Depression Scale.
